# What Works? Toward a New Classification System for Mental Health Supported Accommodation Services: The Simple Taxonomy for Supported Accommodation (STAX-SA)

**DOI:** 10.3390/ijerph15020190

**Published:** 2018-01-24

**Authors:** Peter McPherson, Joanna Krotofil, Helen Killaspy

**Affiliations:** Division of Psychiatry, University College London (UCL), London W1T 7NF, UK; j.krotofil@ucl.ac.uk (J.K.); h.killaspy@ucl.ac.uk (H.K.)

**Keywords:** supported accommodation, mental health, classification, taxonomy

## Abstract

Inconsistent terminology and variation in service models have made synthesis of the supported accommodation literature challenging. To overcome this, we developed a brief, categorical taxonomy that aimed to capture the defining features of different supported accommodation models: the simple taxonomy for supported accommodation (STAX-SA). Data from a previous review of existing classification systems were used to develop the taxonomy structure. After initial testing and amendments, the STAX-SA and an existing taxonomy were applied to 132 supported accommodation service descriptions drawn from two systematic reviews and their performance compared. To assess external validity, the STAX-SA was distributed to a sample of supported accommodation managers in England and they were asked to use it to classify their services. The final version of the STAX-SA comprised of five supported accommodation ‘types’, based on four domains; *Staffing location*; *Level of support*; *Emphasis on move-on*; and *Physical setting*. The STAX-SA accurately categorized 71.1% (*n* = 94) of service descriptions, outperforming the comparison tool, and was not affected by publication date or research design. The STAX-SA effectively discriminated between ‘real world’ service models in England and 53.2% (*n* = 17) of service managers indicated that the taxonomy was ‘*Very effective*’ or ‘*Extremely effective*’ in capturing key characteristics of their service. The STAX-SA is an effective tool for classifying supported accommodation models and represents a promising approach to synthesizing the extant effectiveness literature. The authors recommend the development of reporting guidelines for future supported accommodation publications to facilitate comparison between models.

## 1. Introduction

The need to effectively categorise and map mental health services has been a research priority for some years. International variation in health systems and service configurations has made comparisons difficult; establishing a standardised methodology for classifying services, researchers have argued, would provide a “*rational base for service planning*” ([1], p. 17). Indeed, projects such as the World Health Organization Assessment Instrument for Mental Health Systems (WHO-AIMS) [2], the European Service Mapping Schedule (ESMS) [1] and Description and Evaluation of Services and Directories in Europe-Long Term Care (DESDE-LTC) [3] have provided significant advances in this area, providing empirically-derived descriptions of mental health systems and services, thus facilitating comparisons of effectiveness and cost-effectiveness.

An analogous issue has faced researchers of supported accommodation services. These services provide residential, community-based support for individuals with severe mental health problems and/or complex needs and were originally developed during the deinstitutionalisation processes of the 1980s and 1990s. Services evolved in response to local economic, political and governance factors and, as a result, there is now wide variation in service structures, ranging from 24-h, high-support, residential care settings, through to fully independent accommodation with contact from visiting staff. These models differ widely in terms of target population, physical structure, the number of places provided, staffing levels, staffing qualifications, medication management, recovery focus, move-on policies and integration with local statutory services [4,5]. Compounding this problem, terminology and definitional issues are also widespread; a recent review of housing services for people with mental health problems identified 307 unique terms for supported accommodation across 400 articles [6]. Currently, there are no agreed methods for reporting supported accommodation models in the literature.

With over 30 years of research in the field, model variation and inconsistent service definitions represent significant obstacles to the synthesis of effectiveness data. Indeed, the majority of systematic reviews in this field have been unable to effectively compare service models (e.g., [7]) thus limiting the utility of the findings, leaving researchers and policy makers with little comparative evidence to inform system-level decisions. As stated by Fakhoury and colleagues [8], “*(The) diversity of models in relation to supported housing and inconsistent use of terminology…makes it difficult to compare outcomes or processes*” (p. 311). What is required is a classification system that differentiates service models based on critical characteristics: a taxonomy. A taxonomy is defined as “*a system for classifying multifaceted, complex phenomena according to common conceptual domains and dimensions*” ([9], p. 1765), which can improve both the quality and impact of research by identifying elements of an intervention that might be associated with service user outcomes (e.g., staffing levels), enhance replication and follow-up of studies by improving intervention descriptions and facilitate exploration and synthesis of existing effectiveness data [10]. These benefits are particularly salient for supported accommodation research.

There have been few attempts to address these issues systematically. Fidelity scales for Housing First programs, a model of supported housing for homeless individuals with mental health problems, have been developed [11] and, highlighting the importance of model clarity, research has indicated a relationship between program fidelity and service user outcomes [12,13]. Housing first, however, is a specific approach to supported housing and, as noted, there are countless variations of supported accommodation services currently in operation internationally. Typically, where a classification system is required to capture a more diverse range of services, researchers have used bespoke, non-validated approaches, applying them within a single paper; these descriptions are generally designed to describe services within a specific locality [14] or country [15] and may have limited applicability outside these contexts. Whilst this approach can be useful within an article, it does little to assist in making sense of the literature more broadly. In a noteworthy exception, Siskind and colleagues [16] reviewed existing supported accommodation classification systems (*N* = 18) and, using a structured approach, arranged classification features into a new domains-based taxonomy, comprised of 17 dimensions across four domains: *Duration*; *Patient characteristics*; *Housing characteristics*; and *Service characteristics*. Although comprehensive, the taxonomy has not been used widely in the supported accommodation literature.

We therefore aimed to develop and validate a simple taxonomy for the classification of supported accommodation services, assess and compare the performance of the new taxonomy with the domains-based taxonomy developed by Siskind and colleagues [16] and then examine the external validity of the new taxonomy. It was anticipated that the resulting, validated classification system could be used to synthesise the existing effectiveness research, comparing outcomes across models, thus improving our understanding of what works and for whom.

## 2. Materials and Methods

### 2.1. Materials

#### Domains-Based Taxonomy

To assess the performance of the new classification tool, the domains-based taxonomy of supported accommodation (DTSA) [16] was used as a comparator. The DTSA uses 17 data points across four domains to classify supported accommodation services: 1. *Duration* (Duration of tenure); 2. *Patient characteristics* (Level of need; Readiness to receive treatment; Sobriety required; Subpopulations); 3. *Housing characteristics* (Structure; Location; Proximity to mental health services; Lease); 4. *Service characteristics* (Staffing location; Staffing duration; Service linkage; Staff qualifications; Intensity of support; Service flexibility; Patient choice in housing; Shift in locus of control to patient).

### 2.2. Method

#### 2.2.1. Development

A simple, categorical taxonomy was developed to enable classification of supported accommodation service descriptions from the literature. To ensure the core features of service models were captured, the five most commonly reported dimensions from Siskind and colleagues’ [16] review of 18 existing classification systems were identified: Service duration (length of time service users are involved with the program; 18/18); Housing structure (physical layout of the service; 17/18), Staffing duration (number of hours of staff support; 17/18), Intensity of support (level of involvement required of service users to attend therapeutic groups and/or rehabilitation services; 13/18) and Staffing location (staff based onsite or off-site; 13/18). These elements were reviewed by Peter McPherson, Joanna Krotofil and Helen Killaspy and a draft taxonomy structure developed. The draft structure was then reviewed by an expert panel, comprised of members of the ‘*Quality and Effectiveness of Supported Tenancies for people with mental health problems (QuEST)*’ project management group (a national programme of research into mental health supported accommodation funded by the National Institute of Health Research [NIHR], Ref. RP-PG-0610-10,097), and, based on feedback, amendments were made.

The resultant, initial version of the new taxonomy comprised five supported accommodation ‘Types’, based on variation across five domains; *Staffing location*; *Level of support*; *Recovery-focus*; *Emphasis on move-on*; and *Physical setting* (e.g., Type 1: staff on-site, high support, limited focus on recovery, limited emphasis on move-on, congregate setting). The tool was designed to utilize information extracted from a service description to classify a service as Types 1–5; where data relating to one of the domains is not provided/available, a service cannot be classified.

#### 2.2.2. Initial Testing and Refinement

Service descriptions from retained papers from two recent systematic reviews were used to assess the utility of the new taxonomy; a quantitative review of mental health and psychosocial outcomes associated with supported accommodation [7] and a qualitative review of service user experiences of supported accommodation [17]. The search strategies are described elsewhere, however, to summarize, an electronic search was conducted (January 2015, updated June 2017) using 10 databases; terms and concepts relating to ‘mental illness’, ‘supported accommodation’ and key outcomes, such as quality of life, housing retention and social functioning, were combined with MeSH terms, subject headings or thesaurus terms (depending on database). Limits relating to age (18–65 years) and publication date (>1990) were applied. Hand searches, forward-backward snowballing and recommendations from an expert panel were also used to ensure comprehensiveness. Studies that reported outcomes on individuals with a primary mental health diagnosis were retained. Studies focusing on service users with a primary diagnosis of dementia, learning disability, personality disorder, substance misuse, eating disorder or physical disability were excluded. Studies with an explicit focus on mental health-substance misuse dual diagnosis populations, or those that included a sample with fewer than 50% of participants with a mental health problem were also excluded.

Initially, both the new taxonomy and DTSA were used to classify service descriptions from the quantitative papers. As the new, categorical taxonomy cannot classify a service description where relevant information is not reported, we inspected patterns of missing data across each of the domains (i.e., reported vs. not reported). To improve the performance of the new taxonomy and ensure it was structured according to what is reported in the literature, the patterns of missing data were used to guide subsequent amendments to the structure of the new taxonomy. The amended taxonomy was then reapplied to the quantitative papers to ensure that the changes improved its ability to classify service descriptions.

#### 2.2.3. Final Testing

The final version, named the Simple Taxonomy for Supported Accommodation (STAX-SA) and DTSA were then applied to all valid service descriptions in the retained papers from both systematic reviews. The taxonomies were compared on their ability to effectively categorise the service descriptions using percentage scores. 

#### 2.2.4. Validation: Publication Date and Research Design

To examine whether the performance of the STAX-SA was affected by publication date or research design—for example, with reporting practices changing over time, or qualitative papers having more space to describe services—a series of Chi square tests, comparing the performance of the STAX-SA (service description classified vs. not classified) and publication date (1990–1999; 2000–2009; 2010–2017) and research design (quantitative vs. qualitative) were conducted.

#### 2.2.5. External Validity

To assess the external validity of the STAX-SA, that is, how effective the tool was in differentiating between ‘real world’ supported accommodation service models, managers from a nationally representative sample of supported accommodation services (floating outreach, supported housing and residential care) were contacted by Peter McPherson and Joanna Krotofil (see [18] for original sampling methodology). Services were defined as follows: Residential care provides time unlimited, residential-based support to service users with high needs and offers communal facilities and 24-h staffing; Supported housing provides tenancies in shared or individual self-contained apartments, with staff based on site up to 24-h per day, offering support to increase independence and promote move-on; and, floating outreach services provide time-limited, support to higher-functioning service users living in self-contained, individual tenancies, with visits from support staff based off site. Supported accommodation managers were asked to report the number of services they were responsible for and instructed to categorise each service according to the new taxonomy. For each service categorized, they were also asked how effectively the selected service type captured the key characteristics of the service, using a 5-point Likert scale (1 = ‘*Not at all*’, 5 = ‘*Extremely*’).

## 3. Results

### 3.1. Initial Testing and Refinement

Testing and refinement procedures were conducted using service descriptions from papers retrieved through the systematic reviewed described above. The initial sample from the systematic reviews consisted of 165 papers (quantitative: *n* = 115; qualitative: *n* = 50), however 64 papers were excluded from the current validation procedure (see Table 1). Non-English language papers were excluded to ensure faithful application of the taxonomies. The final pool consisted of 132 service descriptions across 101 papers (quantitative: 95 service descriptions across 72 papers; qualitative: 37 service descriptions across 29 papers) [19,20,21,22,23,24,25,26,27,28,29,30,31,32,33,34,35,36,37,38,39,40,41,42,43,44,45,46,47,48,49,50,51,52,53,54,55,56,57,58,59,60,61,62,63,64,65,66,67,68,69,70,71,72,73,74,75,76,77,78,79,80,81,82,83,84,85,86,87,88,89,90,91,92,93,94,95,96,97,98,99,100,101,102,103,104,105,106,107,108,109,110,111,112,113,114,115,116,117,118]. The majority of studies were American (*n* = 34), with smaller numbers investigating supported accommodation services in Canada (*n* = 17), Australia (*n* = 11), the UK (*n* = 10), Sweden (*n* = 4), Denmark (*n* = 3), Hong Kong (*n* = 3), Israel (*n* = 3), Italy (*n* = 3), Japan (*n* = 3) and Norway (*n* = 2). Single papers from Albania, Belgium, Germany, Greece, Holland, India, Spain and Taiwan were also included (*n* = 8).

To assess inter-rater reliability, Joanna Krotofil and Peter McPherson applied the new taxonomy to nine service descriptions across four randomly selected papers. Discrepancies occurred on 2/45 data points, equalling an error rate of 4.4%. For the initial testing procedure, both taxonomies were then applied to all service descriptions reported in quantitative papers (*n* = 95). Inspection demonstrated that the new taxonomy effectively classified 42.1% (*n* = 40) of service descriptions. Missing data for each domain of the STAX-SA was then checked to identify potential areas for amendment (see Table 2).

Most missing data was in the Recovery domain; 30.5% (*n* = 29) of service descriptions did not report on this variable. This domain was therefore removed from the taxonomy to improve its performance. The amended version of the new taxonomy was then reapplied to the service descriptions. Data are presented in Table 3.

The adjustment improved the ability of the taxonomy to categorise service descriptions, with 71.6% (*n* = 68) of services able to be classified with the amended model. Of the 27 descriptions that could not be classified, 22 (81.5%) had inadequate descriptions of the service and 5 (18.5%) did not ‘fit’ with the STAX-SA descriptions (i.e., the description of services across the four domains did not correspond to a ‘Type’).

The DTSA was also inspected for missing data. No service description was able to be classified across all 17 sub-domains; the median was 9/17, with a range of 1–16. Missing data for the sub-domains are presented in Table 4.

The decision to remove the Recovery domain from the new taxonomy was supported by these findings; the DTSA sub-domains ‘Patient choice in housing’ and ‘Shift in locus of control to patient’ both reflect the recovery focus of a service [16] and demonstrated the second and fifth highest rates of missingness across the 17 dimensions.

### 3.2. Final Testing: STAX-SA

**Figure 1:**
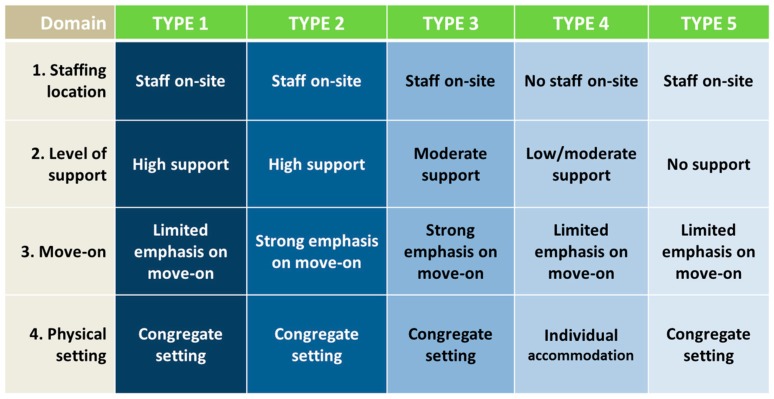
Final structure of the STAX-SA.

The final structure of the new taxonomy, the STAX-SA, is presented in Figure 1.

Guidance for completing each domain of the STAX-SA and the available response options, is presented in Table 5.

Both the STAX-SA and the DTSA were applied to the full sample of service descriptions (*n* = 132). The STAX-SA was able to categorise 71.1% (*n* = 94) of service descriptions (see Table 6). Of the 38 service descriptions that could not be classified, 29 (76.3%) had insufficient description of the service and 9 (23.7%) did not fit in the new taxonomy.

Comparing this to the DTSA, no service could be fully described (i.e., 17/17 dimensions completed). Overall, 45 service descriptions (34.1%) could be categorized on at least 8/17 dimensions (range: 1–16; median: 9/17). Individual dimensions with the highest rates of completion were: Location: *n* = 119 (90.2%); Staff location: *n* = 117 (88.6%); Duration of tenure: *n* = 114 (86.4%); Housing structure: *n* = 111 (84.1%); and, Level of need: *n* = 100 (75.8%).

### 3.3. Publication Date and Research Design

No significant association between publication date and the ability of the STAX-SA to classify services was identified, X^2^ = 0.046, *p* = 0.977. See Table 7 for descriptives. Similarly, there was no statistically significant difference in the classifiability of quantitative (*n* = 68/95) or qualitative papers (*n* = 26/37) (X^2^ = 0.022, *p* = 0.881).

### 3.4. External Validity

Service managers (*n* = 19) used the STAX-SA to classify 32 supported accommodation services in England. Data, presented according to service model and STAX-SA type, are provided in Table 8.

In total, 53.2% (*n* = 17) of service managers indicated that the taxonomy was ‘*Very effective*’ or ‘*Extremely effective*’ in capturing the key characteristics of the service (Mean score (X/5): RC = 4.25, SH = 3.24, FO = 3.91). None indicated that it was ‘*Not effective at all*’.

## 4. Discussion

The current study aimed to develop a simple taxonomy to effectively classify supported accommodation services. The STAX-SA represents a promising approach and is one of the few validated classification systems available in the sector.

### 4.1. Application to the Literature

The STAX-SA was effective in classifying service descriptions from across the supported accommodation literature. Its use is not limited to a particular country or locality; by utilizing the neutral term ‘Types’, the STAX-SA aims to avoid the pitfalls associated with confused terminology in this area. In circumstances where the STAX-SA was unable to classify individual service descriptions, this was predominately due to poor reporting within articles, as opposed to a ‘bad fit’ with the categories, suggesting that the tool captures the majority of model configurations described in the literature. Furthermore, the tool was able to classify service descriptions dating back to 1990 and was not influenced by publication date or research methodology. Conversely, the DTSA appeared to be too comprehensive to apply to supported accommodation models described in the literature; no service descriptions reported data covering all 17 subdomains from the taxonomy. Validating the structure of the STAX-SA, however, the sub-domains of the DTSA with the highest rates of completion mapped broadly onto the domains of the new tool (Location [DTSA] = Physical setting [STAX-SA]; Staff location [DTSA] = Staffing location [STAX-SA]; Duration of tenure [DTSA] = Move-on [STAX-SA]; Housing structure [DTSA] = Physical setting [STAX-SA]; Level of need [DTSA] = Level of support [STAX-SA]).

The comparison between the STAX-SA and the DTSA raises the question: when designing a classification system, should we aim for comprehensiveness, or utility? How we respond to this question will be informed by the purpose for which the classification system is to be used. When comparing effectiveness data or synthesizing the supported accommodation literature more broadly (an essential and overdue task), a reductive approach to classification may be most beneficial; indeed, the simplicity and brevity of the STAX-SA makes it particularly useful for this application. As papers typically provide limited information when describing supported accommodation settings, attempting to utilize a more complex tool, such as the DTSA, will be problematic. However, even when detailed descriptive information is available, the complexity of the DTSA would make comparison between models extremely difficult; as stated by Siskind and colleagues [16] “*With multiple permutations of dimensions, identification of identical supported housing programs for comparison will be challenging. An increase in precision carries with it a loss of simplicity*” (p. 893). The comprehensiveness of the DTSA makes it most suitable for in vivo applications, such as describing program elements when conducting service evaluations. The STAX-SA and DTSA appear to be suited to different but complementary, purposes.

### 4.2. External Validity

Importantly, the STAX-SA can differentiate between ‘real world’ supported accommodation models. As anticipated, residential care services, time unlimited, 24-h staffed communal settings, were predominately classified by service managers as Type 1 and floating outreach services, where service users are visited by support staff in their own, permanent, self-contained tenancy, were predominately classified as Type 4. Supported housing services, however, were less clearly differentiated by the STAX-SA, with managers classifying these services across both Type 2 and Type 3. The sole difference between Type 2 and 3 is the level of support (high vs. moderate) and the observed split likely represents the large variation within supported housing models in the UK; while the majority of supported housing services have staff based onsite, the level of service user need and the level of support provision varies greatly. Some services offer 24-h staff support to individuals with more complex needs, whilst others, that support service users with higher levels of independent living skills, have staff onsite for less than 4 h per day [119]. These differences may also explain why supported housing managers scored the STAX-SA lowest when asked how effective the tool was in capturing the key characteristics of their service.

### 4.3. Strengths and Limitations

This study has a number of strengths. Firstly, the STAX-SA was developed using data from an existing review of classification features [16] and refined according to current reporting practices (e.g., what is reported in the literature). A thorough validation procedure was carried out, assessing the applicability of the taxonomy against 132 service descriptions across 101 papers. Further, the tool is brief, easily administered and able to classify most models based on service descriptions in the literature. Finally, based on our validation procedures, the STAX-SA appears to be applicable to ‘real world’ services, can discriminate between service types in England and, importantly, service managers’ report that it effectively captures the key characteristics of services.

In spite of the identified strengths of the STAX-SA, a number of limitations must be acknowledged. First, is its inability to capture variation within service models; a categorical taxonomy is, by definition, reductive and cannot accommodate service configurations that differ from those specified. This may be particularly pertinent to the supported accommodation sector where local pressures have resulted in unique service models. For example, one service model, the evolving consumer household [46], was unable to be categorized by the STAX-SA; this model provides permanent, shared, community housing to a group of service users, where staff, who are based on-site, gradually reduce their presence over time. It should be acknowledged, however, that examples such as these are relatively rare in the literature and, as described previously, the majority of service descriptions that could not be classified by the STAX-SA were due to insufficient information, as opposed to unique configurations across the four domains. In addition, the STAX-SA may require further validation. Although the STAX-SA was designed primarily to synthesize the relevant effectiveness literature and the primary validation approach utilized service descriptions in studies from 19 different countries, the external validation procedure was conducted solely with English supported accommodation services; to further support the applicability of the taxonomy, it would be useful to replicate the external validity procedure described here with service managers in countries outside of England. This process would address any underlying concerns that the STAX-SA is appropriate only to the British context. Future validation procedures may also seek to consider the tool’s applicability to rural vs. urban services. If undertaken, these procedures could be improved by obtaining further, open-response explanations of ratings of effectiveness of the STAX-SA; in the current study, we did not investigate the reasons for manager ratings of the tool and were, therefore, unable to interpret these ratings in order to address concerns or weaknesses of the tool.

### 4.4. Future Directions

Although the STAX-SA demonstrated the ability to effectively categorise over 70% of service descriptions, a significant proportion could not be classified due to a lack of descriptive information; if sufficient detail had been reported, the tool could potentially have categorized over 93% (123/132) of these service descriptions. We therefore recommend that researchers and journal editors work together to establish an agreed set of reporting guidelines when publishing articles about supported accommodation. Just as the CONSORT guidelines [120] aim to improve the transparency of RCTs, improved reporting in supported accommodation studies would improve both the quality and impact of research in this field. Detailed and clear descriptions of the critical features of supported accommodation models would allow replication studies, enable ongoing exploration and comparison of effectiveness data and, importantly, allow research evidence to be translated into service development and improved outcomes for service users [121]; these benefits are evidenced within the supported accommodation field by the established link between model fidelity and outcomes in Housing First programs [12,13]. Specifically, we recommend that, at a minimum, articles report on the four domains of the STAX-SA—*Staffing location*, *Level of support*, *Move-on* and *Physical setting*—however, consensus is necessary; future studies identifying the essential elements of supported accommodation models that would shape these guidelines, perhaps using the Delphi method, would be welcomed. It is likely that any agreed guidelines would include elements beyond those included in the STAX-SA, such as whether the lease is held by the service user or support-provider, the level of integration between accommodation and mental health services, service user tenancy rights and the philosophy/culture of the service (e.g., recovery-orientation). An alternative approach would be to adapt an existing checklist based on published guidance for reporting public health interventions (e.g., [122,123]).

As with all taxonomies, the STAX-SA should be subject to amendment over time; flexibility is necessary to capture and reflect any significant changes in the target phenomena. With the aim of developing a simple taxonomy that could be applied to the existing literature, it was, initially, necessary to be reductive when developing the STAX-SA. However, as the quality of supported accommodation research improves and the detail in which these complex interventions are described increases, the tool should be adapted to accommodate these advances. For example, the decision was made to omit the *Recovery* domain from the final version of the STAX-SA; although this step vastly improved the applicability of the tool, the importance of the concept of personal recovery, within both accommodation services and in the broader mental health literature, will be reflected in descriptions of service models and may necessitate its inclusion at a later point in time. Similarly, as the sector moves increasingly towards support models based on individual, rather than congregate, settings, the structure of the STAX-SA may need to be amended to reflect this. During our validation procedures only one incidence of a Type 5 service was identified in the literature, suggesting that this service model is relatively uncommon; therefore, it may be useful, in later versions of the STAX-SA, to remove this service type in order to improve the appropriateness of the tool.

## 5. Conclusions

The STAX-SA is a simple, categorical taxonomy that can effectively classify different supported accommodation models described in the literature, based on defining characteristics. The development of this tool was necessary to make sense of the existing literature base, which is beleaguered by inconsistent terminology and broad variation in service models. The STAX-SA represents a promising advancement in this area, though further validation work is required to ensure the tool is applicable to the majority of international service models. In order to progress research in the area of supported accommodation, we recommend that journals agree specific reporting guidelines to ensure adequate and comparable descriptions of supported accommodation models.

## Figures and Tables

**Table 1:** Excluded papers.

Reason	*n*
Multiple sites, with limited service descriptions	40
Mixed method design (assessed in quantitative extraction only)	8
Non-English language	7
Database studies	4
No description of supported accommodation setting	4
Paper inaccessible	1
Total	64

**Table 2:** Missing data according to domain.

Domain	Completed: *n* (%)	Missing: *n* (%)
Staffing location	84 (88.4%)	11 (11.6%)
Level of support	86 (90.5%)	9 (9.5%)
Recovery	66 (69.5%)	29 (30.5%)
Move-on	79 (83.2%)	16 (16.8%)
Physical setting	87 (91.6%)	8 (8.4%)

**Table 3:** Performance of initial and amended, models.

Service Type	Initial Model: *n* (%)	Amended Model: *n* (%)
Type 1	6 (6.3%)	22 (23.2%)
Type 2	11 (11.6%)	13 (13.7%)
Type 3	0 (0.0%)	3 (3.2%)
Type 4	22 (23.2%)	29 (30.5%)
Type 5	1 (1.1%)	1 (1.1%)
Unable to assign type	55 (57.9%)	27 (28.4%)
Total	95 (100.0%)	95 (100.0%)

**Table 4:** DTSA: Missing data according to sub-domain.

Domain	Sub-Domain	Completed (%)	Missing (%)
Duration	Duration of tenure	80 (84.2%)	15 (15.8%)
Patient characteristics	Level of need	70 (73.7%)	25 (26.3%)
	Readiness to receive treatment	33 (34.7%)	62 (65.3%)
	Sobriety required?	16 (16.8%)	79 (83.2%)
	Subpopulations (i.e., women, dual-diagnosis)	94 (98.9%)	1 (1.1%)
Housing characteristics	Structure (congregate vs. individual units)	80 (84.2%)	15 (15.8%)
	Location (cluster vs. scattered site)	83 (87.4%)	12 (12.6%)
	Geographic proximity to mental health services	13 (13.7%)	82 (86.3%)
	Lease (i.e., held by patient or agency)	20 (21.1%)	75 (78.9%)
Service characteristics	Staffing location (onsite vs. off-site)	83 (87.4%)	12 (12.6%)
	Staffing duration	58 (61.1%)	37 (38.9%)
	Service linkage	35 (36.8%)	60 (63.2%)
	Staff qualifications (clinical vs. non-clinical)	41 (43.2%)	54 (56.8%)
	Intensity of support	58 (61.1%)	37 (38.9%)
	Service flexibility (i.e., variation in support)	60 (63.2%)	35 (36.8%)
	Patient choice in housing	16 (16.8%)	79 (83.2%)
	Shift in locus of control to patient	29 (30.5%)	66 (69.5%)

**Table 5:** Domains and response guidance for the STAX-SA.

Domain	Guidance	Response Options
Staffing location	Are support staff based on-site (e.g., at the accommodation) or off-site?	1. Staff on-site 2. No staff on-site
Level of support	Level of support should reflect frequency, nature and intensity of support (including staffing duration) and the level of service user need (e.g., for personal care, medication management).	1. High support 2. Moderate support 3. Low support 4. No support
Move-on	How much emphasis is placed on service users moving to another physical setting after demonstrating clinical improvement, or after a set period of time (e.g., time-limited tenancies vs. open-ended tenancies/permanent housing)?	1. Strong emphasis on move-on 2. Limited emphasis on move-on
Physical structure	Congregate setting = Shared with other mental health service users. Communal facilities.	1. Congregate setting 2. Individual accommodation
	Individual accommodation = Generic, independent community housing (not mental health specific)	

**Table 6:** Performance of STAX-SA.

Service Type	Final Model: *n* (%)
Type 1	31 (23.5%)
Type 2	19 (14.4%)
Type 3	4 (3.0%)
Type 4	39 (29.5%)
Type 5	1 (0.8%)
Unable to assign type	38 (28.8%)
Total	132 (100.0%)

**Table 7:** STAX-SA classification according to publication date.

	Year: 1990–1999	Year: 2000–2009	Year: 2010–2017	Total
Classified	29	34	31	94
Not classified	12	13	13	38
Total	41	47	44	132

**Table 8:** Service manager STAX-SA classifications according to supported accommodation model.

Service Type	Type 1	Type 2	Type 3	Type 4	Type 5
Residential care (*n* = 4)	3 (75.0%)	1 (25.0%)	-	-	-
Supported housing (*n* = 17)	1 (5.9%)	7 (41.2%)	9 (52.9%)	-	-
Floating outreach (*n* = 11)	-	-	2 (18.2%)	9 (81.8%)	-

## Abbreviations

The following abbreviations are used in this manuscript:

STAX-SA	Simple Taxonomy for Supported Accommodation
DTSA	Domains-Based Taxonomy for Supported Accommodation
WHO-AIMS	World Health Organization Assessment Instrument for Mental Health Systems
ESMS	the European Service Mapping Schedule
DESDE-LTC	Description and Evaluation of Services and Directories in Europe-Long Term Care
QUEST	Quality and Effectiveness of Supported Tenancies for people with mental health problems
NIHR	National Institute for Health Research
UK	United Kingdom
MH	Mental Health
RC	Residential Care
SH	Supported Housing
FO	Floating Outreach
CONSORT	Consolidated Standards of Reporting Trials
RCT	Randomized controlled trial
